# Complete Blood Cell Count-Derived Biomarkers and Clinical Studies: Is It Time for New Reporting Criteria? Comment on Anand et al. Utility of Red Cell Distribution Width (RDW) as a Noninvasive Biomarker for the Diagnosis of Acute Appendicitis: A Systematic Review and Meta-Analysis of 5222 Cases. *Diagnostics* 2022, *12*, 1011

**DOI:** 10.3390/diagnostics12102329

**Published:** 2022-09-27

**Authors:** John L. Frater, M. Yadira Hurley

**Affiliations:** 1 Department of Pathology and Immunology, Washington University, St. Louis, MO 63110, USA; 2 Department of Pathology, Saint Louis University, St. Louis, MO 63104, USA

We read with great interest the article entitled “Utility of Red Cell Distribution Width (RDW) as a Noninvasive Biomarker for the Diagnosis of Acute Appendicitis: A Systematic Review and Meta-Analysis of 5222 Cases” by S. Anand et al. which has been recently published in *Diagnostics* [1]. We congratulate the authors for their work, which contributes to the burgeoning number of studies that evaluate the clinical utility of complete blood cell count (CBC)—derived analytes in risk stratification and outcome. We would like to offer the following remarks regarding the RDW and its reporting in clinical studies, which we hope will add useful context for the readership of *Diagnostics* who are considering the use of CBC-derived biomarkers for patient care purposes.

It is important to note that CBC parameters may be affected by several preanalytical and analytical phase variables which could potentially bias results. For the RDW, these include ambient temperature, time between phlebotomy and analysis, anticoagulant type, and storage/transport conditions [2]. In addition, there are known problems with standardization of the RDW across different instrument platforms [3], and in the absence of an internationally recognized standard, it is difficult to arrive at a solution to this issue. We therefore reviewed the studies used by Anand et al. [1] and extracted the information regarding these variables, the results of which are summarized in Table 1. There is an obvious lack of transparency regarding these potentially important variables, with the vast majority of studies providing no information about temperature, time between phlebotomy and analysis, type of anticoagulant, and storage/transport conditions. In addition, studies that used CBC data collected from multiple sites may have introduced bias due to nonuniform processing and analysis of specimens. For example, studies that used outpatient samples as a control group may have included data from specimens collected remotely over the course of the workday and analyzed several hours after phlebotomy.

We note the high levels of heterogeneity reported by Anand et al. in all their subset analyses, with I^2^ scores ranging from 92% to 99% [1]. Although we agree with Anand et al. that aspects of the study designs of these papers such as inclusion criteria and control group selection likely contributed to the “negative” results of their analysis, we hypothesize that lack of control for preanalytical and analytical phase variables in at least some of these studies may also be of importance.

We therefore note that there are obvious issues with the papers cited by Anand et al. regarding the reporting of critical preanalytical and analytical phase variables. The clear and transparent reporting of this information in clinical studies is of obvious importance to readers since it allows them to determine the degree to which the findings of these publications can be applied to their clinical practice. In the era of evidence-based medicine, assessments of quality of primary studies such as the United States Preventative Services Task Force, Downs and Black, and the Newcastle-Ottawa scale are important constituents of the Preferred Reporting Items for Systematic Reviews and Meta-Analyses (PRISMA) checklist and can provide useful information about the reporter quality of the studies used in systematic reviews and meta-analyses [4]. Although the shortcomings of methodological quality and reporting of laboratory data used in clinical studies have been long recognized [5,6,7], a solution to this issue has not yet been widely applied. Moreover, the Standards for Reporting of Diagnostic Accuracy Studies (STARD) criteria, which were implemented to improve the quality of reporting of diagnostic test accuracy studies, are underutilized in the laboratory medicine literature [7,8] and do not define criteria for the reporting of preanalytical and analytical phase variables [9]. In view of the now-widespread use of laboratory data such as CBC-derived analytes in clinical research, there is now a clear need for improvement in the reporting quality of these biomarkers. 

In closing, there is a clear need for greater transparency in clinical studies that use CBC-derived data with regard to potential preanalytical and analytical phase biases, and the current systems that were created to address reporting criteria do not adequately address this problem. We thank Anand et al. for their contribution to the literature on the use of the RDW for clinical care and we hope that these additional comments add useful context to the discussion of this important topic.

## Figures and Tables

**Table 1:** Summary of reporting of preanalytical and analytical phase data from the studies used by Anand et al. [1].

Paper [Ref]	Setting	Age	Study and Control Group(s)	Temperature	Time	Anticoagulant	Storage	Instrumentation
Acar [46]	Surgery, ED	Adult	AA, Renal colic, Normal adults (OP)	RT	NR	Na Citrate	RT	Pentra DF Nexus (Hariba)
Antic [40]	Surgery	Children	Complicated AA, Uncomplicated AA, nonspecific abdominal pain	NR	NR	NR	NR	Advia 2120 (Siemens)
Boshnak [3]	Surgery	Adult	Uncomplicated AA, normal appendix	NR	<1 h	K3 EDTA	NR	Sysmex XT 1800 (Sysmex)
Bozlu [32]	Surgery	Children	Appendectomy, Normal children (OP)	NR	NR	NR	NR	NR
Daldal [42]	Surgery	Adults	Appendix diameter ≥ 6 mm, Appendix diameter ≤ 6 mm	NR	NR	NR	NR	NR
Dinc [38]	Surgery	Adults	Uncomplicated AA, Perforated AA, normal appendix	NR	NR	NR	NR	CoulterLH780 (Beckman Coulter)
Haghi [30]	Surgery	Adults	AA, normal appendix	NR	NR	NR	NR	NR
Maghsoudi [43]	Surgery	Adult	AA, normal appendix	NR	NR	NR	NR	NR
Narci [31]	Surgery	Adult	AA, healthy adults	NR	NR	NR	NR	Cell-Dyne 3700 (Abbott)
Sengul [39]	Surgery	Children	Complicated AA, Uncomplicated AA, normal appendix	NR	NR	NR	NR	NR
Sonmez [47]	Surgery, ED	Adults	AA, renal colic	NR	NR	NR	NR	XN 10 (Sysmex)
Tanrikulu [45]	Surgery, Other sites	Adults	AA, Normal adults (other sites), including OP)	NR	NR	NR	NR	NR
Tartar [37]	Surgery	Children	Complicated AA, Uncomplicated AA, normal appendix	NR	NR	NR	NR	NR
Toktas [41]	Surgery	Adults	AA, Normal adults (OP)	NR	NR	NR	NR	LH 780 (Beckman-Coulter)
Ulukent [44]	Surgery	Adults	AA, Normal adults (OP)	NR	NR	EDTA	NR	LH 780 (Beckman-Coulter)

## Data Availability

The data presented in this study are available upon request from the corresponding author.

## Abbreviations

Ref—reference as listed by Anand et al.; ED—emergency department; AA—acute appendicitis; OP—outpatient; RT—room temperature; NR—not reported; Na—sodium; K—potassium.
